# Using brain structural neuroimaging measures to predict psychosis onset for individuals at clinical high-risk

**DOI:** 10.1038/s41380-024-02426-7

**Published:** 2024-02-09

**Authors:** Yinghan Zhu, Norihide Maikusa, Joaquim Radua, Philipp G. Sämann, Paolo Fusar-Poli, Ingrid Agartz, Ole A. Andreassen, Peter Bachman, Inmaculada Baeza, Xiaogang Chen, Sunah Choi, Cheryl M. Corcoran, Bjørn H. Ebdrup, Adriana Fortea, Ranjini RG. Garani, Birte Yding Glenthøj, Louise Birkedal Glenthøj, Shalaila S. Haas, Holly K. Hamilton, Rebecca A. Hayes, Ying He, Karsten Heekeren, Kiyoto Kasai, Naoyuki Katagiri, Minah Kim, Tina D. Kristensen, Jun Soo Kwon, Stephen M. Lawrie, Irina Lebedeva, Jimmy Lee, Rachel L. Loewy, Daniel H. Mathalon, Philip McGuire, Romina Mizrahi, Masafumi Mizuno, Paul Møller, Takahiro Nemoto, Dorte Nordholm, Maria A. Omelchenko, Jayachandra M. Raghava, Jan I. Røssberg, Wulf Rössler, Dean F. Salisbury, Daiki Sasabayashi, Lukasz Smigielski, Gisela Sugranyes, Tsutomu Takahashi, Christian K. Tamnes, Jinsong Tang, Anastasia Theodoridou, Alexander S. Tomyshev, Peter J. Uhlhaas, Tor G. Værnes, Therese A. M. J. van Amelsvoort, James A. Waltz, Lars T. Westlye, Juan H. Zhou, Paul M. Thompson, Dennis Hernaus, Maria Jalbrzikowski, Shinsuke Koike, the ENIGMA Clinical High Risk for Psychosis Working Group, Paul Allen, Helen Baldwin, Sabrina Catalano, Michael W. L. Chee, Kang Ik K. Cho, Lieuwe de Haan, Leslie E. Horton, Mallory J. Klaunig, Yoo Bin Kwak, Xiaoqian Ma, Merete Nordentoft, Lijun Ouyang, Jose C. Pariente, Franz Resch, Jason Schiffman, Mikkel E. Sørensen, Michio Suzuki, Sophia Vinogradov, Christina Wenneberg, Hidenori Yamasue, Liu Yuan

**Affiliations:** 1 Center for Evolutionary Cognitive Sciences, Graduate School of Arts and Sciences, The University of Tokyo, Tokyo, Japan; 2 Institut d’Investigacions Biomèdiques August Pi i Sunyer (IDIBAPS), CIBERSAM, Instituto de Salud Carlos III, Universitat de Barcelona, Barcelona, Spain; 3 Max Planck Institute of Psychiatry, Munich, Germany; 4 Early Psychosis: Interventions and Clinical‐detection (EPIC) Lab, Department of Psychosis Studies, Institute of Psychiatry, Psychology & Neuroscience, King’s College London, London, UK; 5 Department of Brain and Behavioral Sciences, University of Pavia, Pavia, Italy; 6 Department of Psychiatric Research, Diakonhjemmet Hospital, Oslo, Norway; 7 Centre for Psychiatry Research, Department of Clinical Neuroscience, Karolinska Institutet & Stockholm Health Care Services, Stockholm County Council, Stockholm, Sweden; 8 KG Jebsen Center for Neurodevelopmental Disorders, University of Oslo, Oslo, Norway; 9 Norwegian Centre for Mental Disorders Research, Institute of Clinical Medicine, University of Oslo, Oslo, Norway; 10 Department of Psychiatry and Behavioral Sciences, Boston Children’s Hospital, Boston, MA USA; 11 Department of Child and Adolescent Psychiatry and Psychology, Institute of Neuroscience, 2017SGR-881, Hospital Clinic Barcelona, Institut d’Investigacions Biomèdiques August Pi i Sunyer (IDIBAPS), Centro de Investigación Biomédica en Red de Salud Mental (CIBERSAM), Universitat de Barcelona, Barcelona, Spain; 12 National Clinical Research Center for Mental Disorders and Department of Psychiatry, the Second Xiangya Hospital of Central South University, Changsha, Hunan China; 13 National Clinical Research Center for Geriatric Disorders, Xiangya Hospital, Central South University, Changsha, Hunan China; 14 Department of Brain and Cognitive Sciences, Seoul National University College of Natural Sciences, Seoul, South Korea; 15 Department of Psychiatry, Icahn School of Medicine at Mount Sinai, New York City, NY USA; 16 Mental Illness Research, Education, and Clinical Center, James J Peters VA Medical Center, New York City, NY USA; 17 Centre for Neuropsychiatric Schizophrenia Research (CNSR), Mental Health Centre Glostrup, Copenhagen University Hospital, Glostrup, Denmark; 18 Department of Clinical Medicine, Faculty of Health and Medical Sciences, University of Copenhagen, Copenhagen, Denmark; 19 Department of Child and Adolescent Psychiatry and Psychology, Institute of Neuroscience, Hospital Clinic Barcelona, Fundació Clínic Recerca Biomèdica, Universitat de Barcelona, Barcelona, Spain; 20 Douglas Research Center; Integrated Program in Neuroscience, McGill University, Montreal, QC Canada; 21 Copenhagen Research Center for Mental Health, Mental Health Center Copenhagen, University of Copenhagen Copenhagen, Copenhagen, Denmark; 22 Department of Psychiatry and Behavioral Sciences, University of California San Francisco, San Francisco, CA USA; 23 San Francisco Veterans Affairs Health Care System, San Francisco, CA USA; 24 Department of Psychiatry and Psychotherapy I, LVR-Hospital Cologne, Cologne, Germany; 25 Department of Psychiatry, Psychotherapy and Psychosomatics, Psychiatric University Hospital Zurich, University of Zurich, Zurich, Switzerland; 26 Department of Neuropsychiatry, Graduate School of Medicine, The University of Tokyo, Tokyo, Japan; 27 The University of Tokyo Institute for Diversity and Adaptation of Human Mind, The University of Tokyo, Tokyo, Japan; 28 The International Research Center for Neurointelligence at The University of Tokyo Institutes for Advanced Study (WPI-IRCN), The University of Tokyo, Tokyo, Japan; 29 Department of Neuropsychiatry, Toho University School of Medicine, Tokyok, Japan; 30 Department of Neuropsychiatry, Seoul National University Hospital, Seoul, South Korea; 31 Department of Psychiatry, Seoul National University College of Medicine, Seoul, South Korea; 32 Division of Psychiatry, University of Edinburgh, Edinburgh, UK; 33 Laboratory of Neuroimaging and Multimodal Analysis, Mental Health Research Center, Moscow, Russian Federation; 34 Department of Psychosis, Institute of Mental Health, Singapore, Singapore; 35 Lee Kong Chian School of Medicine, Nanyang Technological University, Singapore, Singapore; 36 Department of Psychiatry, University of Oxford, Oxford, UK; 37 Douglas Research Center; Department of Psychiatry, McGill University, Montreal, QC Canada; 38 Tokyo Metropolitan Matsuzawa Hospital, Tokyo, Japan; 39 Department for Mental Health Research and Development, Division of Mental Health and Addiction, Vestre Viken Hospital Trust, Drammen, Norway; 40 Department of Youth Psychiatry, Mental Health Research Center, Moscow, Russian Federation; 41 Department of Clinical Physiology, Nuclear Medicine and PET, Functional Imaging, University of Copenhagen Copenhagen, Copenhagen, Denmark; 42 Department of Psychiatry and Psychotherapy, Charité Universitätsmedizin Berlin, Berlin, Germany; 43 Department of Psychiatry, University of Pittsburgh, Pittsburgh, PA USA; 44 Department of Neuropsychiatry, University of Toyama Graduate School of Medicine and Pharmaceutical Sciences, Toyama, Japan; 45 Research Center for Idling Brain Science, University of Toyama, Toyama, Japan; 46 Department of Child and Adolescent Psychiatry, Psychiatric University Hospital Zurich, University of Zurich, Zurich, Switzerland; 47 PROMENTA Research Center, Department of Psychology, University of Oslo, Oslo, Norway; 48 Department of Psychiatry, Sir Run Run Shaw Hospital, School of Medicine, Zhejiang University, Zhejiang, China; 49 Key Laboratory of Medical Neurobiology of Zhejiang Province, School of Medicine, Zhejiang University, Zhejiang, China; 50 Department of Child and Adolescent Psychiatry, Charité Universitätsmedizin Berlin, Berlin, Germany; 51 Institute of Neuroscience and Psychology, University of Glasgow, Glasgow, UK; 52 Early Intervention in Psychosis Advisory Unit for South-East Norway, TIPS Sør-Øst, Division of Mental Health and Addiction, Oslo University Hospital, Oslo, Norway; 53 Department of Psychiatry and Neuropsychology, School for Mental Health and Neuroscience, Faculty of Health Medicine and Life Sciences, Maastricht University, Maastricht, The Netherlands; 54 Maryland Psychiatric Research Center, University of Maryland School of Medicine, Baltimore County, Baltimore, MD USA; 55 Department of Psychology, University of Oslo, Oslo, Norway; 56 Centre for Sleep and Cognition, Yong Loo Lin School of Medicine, National University of Singapore, Singapore, Singapore; 57 Centre for Translational Magnetic Resonance Research, Yong Loo Lin School of Medicine, National University of Singapore, Singapore, Singapore; 58 Imaging Genetics Center, Mark and Mary Stevens Institute for Neuroimaging and Informatics, Keck School of Medicine of USC, University of Southern California, Los Angeles, CA USA; 59 Department of Psychiatry, Harvard Medical School, Cambridge, MA USA; 60 Department of Psychology, University of Roehampton, London, UK; 61 Department of Psychosis Studies, Institute of Psychiatry, Psychology and Neuroscience, King’s College London, London, UK; 62 NIHR Maudsley Biomedical Research Centre, South London and Maudsley NHS Foundation Trust and King’s College London, London, UK; 63 Centre for Translational Magnetic Resonance Research,Yong Loo Lin School of Medicine, National University of Singapore, Singapore, Singapore; 64 Department of Psychiatry, Psychiatry Neuroimaging Laboratory, Brigham and Women’s Hospital, Harvard Medical School, Cambridge, MA USA; 65 Department of Psychiatry, Amsterdam University Medical Centre;NA, Arkin, Amsterdam, The Netherlands; 66 Department of Psychology, University of Maryland, Baltimore, MD USA; 67 Hunan Key Laboratory of Psychiatry and Mental Health, the Second Xiangya Hospital, Central South University, Changsha, China; 68 Magnetic Resonance Imaging Core Facility, Institut d’Investigacions Biomèdiques August Pi i Sunyer, Barcelona, Spain; 69 Clinic for Child and Adolescent Psychiatry, University Hospital of Heidelberg, Heidelberg, Germany; 70 Department of Psychological Science, University of California Irvine, Irvine, CA USA; 71 Center for Neuropsychiatric Schizophrenia Research, CNSR, Mental Health Centre Glostrup, University of Copenhagen, Copenhagen, Denmark; 72 Department of Psychiatry & Behavioral Sciences, University of Minnesota, Minneapolis, MN USA; 73 Department of Psychiatry, Hamamatsu University School of Medicine, Hamamatsu, Japan

**Keywords:** Predictive markers, Schizophrenia

## Abstract

Machine learning approaches using structural magnetic resonance imaging (sMRI) can be informative for disease classification, although their ability to predict psychosis is largely unknown. We created a model with individuals at CHR who developed psychosis later (CHR-PS+) from healthy controls (HCs) that can differentiate each other. We also evaluated whether we could distinguish CHR-PS+ individuals from those who did not develop psychosis later (CHR-PS-) and those with uncertain follow-up status (CHR-UNK). T1-weighted structural brain MRI scans from 1165 individuals at CHR (CHR-PS+, *n* = 144; CHR-PS-, *n* = 793; and CHR-UNK, *n* = 228), and 1029 HCs, were obtained from 21 sites. We used ComBat to harmonize measures of subcortical volume, cortical thickness and surface area data and corrected for non-linear effects of age and sex using a general additive model. CHR-PS+ (*n* = 120) and HC (*n* = 799) data from 20 sites served as a training dataset, which we used to build a classifier. The remaining samples were used external validation datasets to evaluate classifier performance (test, independent confirmatory, and independent group [CHR-PS- and CHR-UNK] datasets). The accuracy of the classifier on the training and independent confirmatory datasets was 85% and 73% respectively. Regional cortical surface area measures-including those from the right superior frontal, right superior temporal, and bilateral insular cortices strongly contributed to classifying CHR-PS+ from HC. CHR-PS- and CHR-UNK individuals were more likely to be classified as HC compared to CHR-PS+ (classification rate to HC: CHR-PS+, 30%; CHR-PS-, 73%; CHR-UNK, 80%). We used multisite sMRI to train a classifier to predict psychosis onset in CHR individuals, and it showed promise predicting CHR-PS+ in an independent sample. The results suggest that when considering adolescent brain development, baseline MRI scans for CHR individuals may be helpful to identify their prognosis. Future prospective studies are required about whether the classifier could be actually helpful in the clinical settings.

## Introduction

The clinical high risk (CHR) paradigm is widely used with the goal of improving early detection of and prevention of psychotic disorders [1]. Individuals are considered at CHR for psychosis if they meet criteria for attenuated positive symptom syndrome (APSS), brief intermittent (limited) psychotic syndrome (BLIPS), and/or genetic risk and deterioration syndrome (GRDS) based on semistructured interviews [2–5]. The CHR state is present in 1.7% of the general population and 19.2% of clinical samples [6]. CHR individuals have a higher risk of developing psychosis (0.15 at 1 year) comparing to healthy controls, the transition risk increased from 0.09 at half years to 0.27 at 4 years [7]. However, most CHR subjects who do not transition to psychosis will continue to meet CHR criteria or experience attenuated psychosis symptoms at follow-up and only 33% will eventually remit [7, 8].

The CHR state, is also associated with alterations in proxy measures of brain structure [9–15]. Previous structural magnetic resonance imaging (MRI) studies reported a progressive decrease in gray matter volume in the medial and superior temporal and medial frontal cortex during the transition period among CHR individuals [14–17]. Gray matter volume continued to decrease several years after disease onset [15, 16, 18]. Cortical surface area (SA) and cortical thickness (CT), which can be extracted using FreeSurfer software [19–21], are also crucial predictors of important life outcome [22] and associated with neurological, psychological, and behavioral traits [23]. SA is strongly correlated with grey matter volume compared to CT, suggesting SA and CT are unique structural features in the grey matter cortex [24, 25]. Recent study indicated that the multivariate architectures with respect to the makeup of the genetic factors were distinct across cortical surface area and thickness [22]. This is in line with the radial unit hypothesis [26] that the expansion of cortical surface area is driven by the proliferation of neural progenitor cells, whereas numbers of neurogenetic division of these cells for thickness [23]. Widespread lower CT has also been identified in cross-sectional MRI data in individuals at CHR in a large-scale pooled analysis of the Enhancing Neuro Imaging Genetics through Meta-Analysis (ENIGMA) CHR Working Group [27]. Among these widespread alterations, frontal cortical and temporal regions (e.g., fusiform, superior temporal, and paracentral) have been relatively consistently associated with CHR status [9–11, 28–30], with these regions also exhibiting lower CT in individuals with established schizophrenia [29]. In addition to regional changes, individuals with CHR, have showed greater neuroanatomical variability in global SA, CT, and subcortical volume compared to HC [31]. Furthermore, longitudinal studies have shown reductions of cortical thickness in the paracentral, superior temporal, and fusiform gyrus have been reported to be associated with psychosis conversion in those at CHR [13, 14, 32]. Recent work has indicated that whole-brain sMRI patterns of schizophrenia forecasted 2-year psychosocial impairments in individuals with CHR [33], suggesting that alterations in brain structure may predict real-life outcomes.

Adolescent development is a crucial time window that is associated with brain-wide changes, including reductions in cortical thickness and volume [34, 35]. Cortical characteristics such as gray matter volume, cortical surface area, and cortical thickness decline by about 10% during adolescence [36]. On the other hand, white matter volume was reported peaking in young adulthood [36]. Since the period from adolescence to early adulthood is a high risk time window for psychosis onset [32], age-related anatomical deviations from typically-occuring declines may hold valuable information to predict later psychosis conversion, especially in frontal and temporal regions that have been implicated in CHR [27, 32, 37–39] and schizophrenia [40–45]. Further, greater brain age deviations were found to be associated with a higher risk for psychosis over time [11, 38]. Importantly, these results suggest that the adolescent brain development pattern of CHR individuals may differ from that of HCs. Indeed, the ENIGMA CHR Working Group has reported that CHR compared to HC participants exhibit altered non-linear age associations with cortical thickness [27], suggesting that cross-sectional between-group differences in sMRI metrics may involve altered adolescent development, trait characteristics associated with psychosis liability, and/or progressive brain pathology around the onset of psychosis [32, 39, 46].

An increasing number of studies have attempted to use (cross-sectional) sMRI data to predict outcome or case-control status. These prior studies show that machine learning approaches are informative for differentiating individuals with schizophrenia from HCs [47–52]. Similar findings were observed in different clinical stages of psychosis, including first episode schizophrenia and CHR individuals [48, 49]. A major limitation, however, is the need for large and diverse sample sizes to establish a well-tuned classifier that also provides generalized predictive performance [12, 53]. Since single sites cannot typically provide the necessary sample sizes [49, 54, 55], multisite consortia data may be advantageous if site effects are adequately accounted for (e.g., via cross-site harmonization procedures) [49, 54, 56]. For example, without harmonization, a prior study failed to build a useful model with multi site data [38]. In the current study, we aimed to investigate whether cross-sectional sMRI data can be used to build a classifier to differentiate the neuroanatomical developmental patterns of HCs relative to participants who later developed a psychotic disorder (CHR-PS+) as biomarkers for future psychosis conversion. As altered developmental processes are implicated in psychosis risk, we considered the potential non-linear effects of age and sex to gain optimal predictive accuracy of trained classifiers.

Here, we combined data from 21 sites harmonized through the ENIGMA CHR Working Group using ComBat [57] to minimize differences related to site-, scanner- and scanning protocols using an Empirical Bayes method. Second, to model non-linear age effects, we fitted generalized additive models (GAMs) [58, 59] to the HC data, and then applied the fitted GAMs to obtain non-linear age- and sex-corrected features for the entire sample [60]. More specifically, we estimated the model in HCs and applied it to individuals at CHR to capture deviations from the expected patterns of physiological aging. As for patients with early-onset psychosis [61] and schizophrenia [41] have been reported to have abnormally low estimated intracranial volume (ICV), all procedures were performed after adjusting the MRI features for effects of ICV. Third, we developed an XGBoost [62] classifier using only HCs and CHR-PS+ to determine deviation in neuroanatomical developmental patterns as potential predictors of future psychosis conversion. Finally, we tested the predictive performance of the classifier with the left-out site data, to avoid the potential for information leakage between the training and test data.

We hypothesized that CHR-PS+ individuals would be distinguishable from HCs based on features derived from structural MRI features, based on the assumption that those CHR individuals who are most likely to convert to psychosis would show the greatest baseline anatomical alterations. Second, we expected our classifier to label individuals at CHR who had *not* developed a psychotic disorder (CHR-PS-) at follow-up, and individuals at CHR who did not complete follow-up visits, resulting in missing information about their transition status (CHR-UNK), as HCs. Third, we expected the classifiers to perform similarly in independent confirmatory datasets, and expected to find associations between classifications and symptom severity.

## Methods

### Participants

We included data from a total of 1165 CHR individuals (144 CHR-PS+, 793 CHR-PS−, and 228 CHR-UNK individuals) and 1029 healthy controls (HCs) from 21 ENIGMA Clinical High Risk for Psychosis Working Group sites (Table 1). As previous study showed that using CHR psychometric instruments to assess the CHR state in clinical samples is associated with an excellent overall prognostic performance [63], we combined two assessments directly as previous studies [27, 31, 64]. CHR status was assessed using the full version of the Comprehensive Assessment of At-Risk Mental States (CAARMS [65]; *n* = 650) or the Structured Interview for Prodromal Syndromes (SIPS [66, 67]; *n* = 799). Site-specific inclusion and exclusion criteria, the available scale scores in premorbid IQ, symptom severity, global functioning, and antipsychotic use at scan are the same as in a prior publication (Supplementary Table S1) [27]. All sites obtained local institutional review board approval prior to data collection. Written informed consent was obtained from every participant, or from the participant’s guardian for participants younger than 18 years. All studies were conducted in accordance with the Declaration of Helsinki [68].

**Table 1:** Demographic characteristics of study participants.

	HC			CHR			CHR-PS+			CHR-PS-			CHR-UNK				
	*N*	Female, No. (%)	Age, mean (SD; range)	*N*	Female, No. (%)	Age, mean (SD; range)	*N*	Female, No. (%)	Age, mean (SD; range)	*N*	Female, No. (%)	Age, mean (SD; range)	*N*	Female, No. (%)	Age, mean (SD; range)	Transition rate,%	Follow-up length,mean (SD)
**Total**																	
Total	1029	438 (43)	22.48 (5.17; 11.30–39.87)	1165	535 (46)	20.78 (4.82; 10.30–39.00)	144	59 (41)	19.85 (4.60; 12.6–35.00)	793	373 (47)	20.83 (4.95; 10.30–39.00)	228	103 (45)	21.19 (4.45; 12.00–34.39)	12.36	18.49 (14.64)
Training	799	342 (43)	22.07 (5.20; 11.30–39.87)	120	52 (43)	19.76 (4.62; 12.6–35.00)	120	52 (43)	19.76 (4.62; 12.6–35.00)	0	NA	NA	0	NA	NA	NA	17.28 (1053)
Test	89	29 (33)	22.07 (5.08; 12.90–39.25)	14	4 (29)	19.96 (3.73; 14.00–26.00)	14	4 (29)	19.96 (3.73; 14.00–26.00)	0	NA	NA	0	NA	NA	NA	15.47 (7.89)
Independent confirmatory	141	67 (48)	25.07 (4.22; 18.00–38.00)	10	3 (30)	20.76 (5.72; 14.90–31.40)	10	3 (30)	20.76 (5.72; 14.90–31.40)	0	NA	NA	0	NA	NA	NA	40.2 (36.71)
Independent group	0	NA	NA	1021	476 (47)	20.91 (4.84; 10.30–39.00)	0	NA	NA	793	373 (47)	20.83 (4.95; 10.30–39.00)	228	103 (45)	21.19 (4.45; 12.00–34.39)	NA	19.15 (15.45)
**Site**																	
Columbia	9	2 (22)	24.35 (4.05; 19.58–33.09)	17	9 (53)	22.89 (4.96; 14.87–30.69)	3	2 (67)	19.40 (7.32; 14.87–27.84)	14	7 (50)	23.63 (4.32; 15.91–30.69)	0	NA	NA	17.65	20.63 (10.32)
Copenhagen	58	29 (50)	24.78 (3.30; 20.00–35.00)	163	86 (53)	24.18 (4.18; 18.00–38.00)	13	5 (38)	23.08 (3.40; 18.00–29.00)	95	54 (57)	24.51 (4.57; 18.00–38.00)	55	27 (49)	23.89 (3.60; 19.00–33.00)	7.98	11.20 (2.05)
CSU	59	25 (42)	21.49 (3.14; 15.00–30.00)	52	24 (46)	19.48 (5.00; 13.00–35.00)	21	12 (57)	19.43 (5.07; 13.00–35.00)	31	12 (39)	9.52 (5.04; 13.00–30.00)	0	NA	NA	40.38	14.65 (7.55)
Glasgow	46	30 (65)	22.80 (3.62; 18.00-32.00)	80	63 (79)	22.24 (4.79; 17.00–34.00)	6	5 (83)	18.50 (1.87; 17.00–22.00)	74	58 (78)	22.54 (4.83; 17.00–34.00)	0	NA	NA	7.50	20.18 (9.85)
Heidelberg	33	16 (48)	15.73 (0.91; 14.00–17.00)	22	13 (59)	15.14 (1.08; 14.00–17.00)	0	NA	NA	18	12 (67)	15.11 (1.02; 14.00–17.00)	4	1 (25)	15.25 (1.50; 14.00–17.00)	NA	9.72 (3.32)
IDIBAPS	54	35 (65)	15.86 (1.63; 11.30-18.30)	74	49 (66)	15.26 (1.69; 10.30–18.10)	17	11 (65)	15.07 (1.38; 12.60–17.20)	42	28 (67)	15.49 (1.90; 10.30–18.10)	15	10 (67)	14.85 (1.34; 12.80–17.00)	22.97	14.40 (5.49)
ISMMS	12	5 (42)	27.94 (3.77; 22.79–34.83)	25	13 (52)	23.39 (5.43; 17.11–34.89)	1	0	26.31 (NA)	12	7 (58)	23.63 (5.46; 17.17–34.89)	12	6 (50)	22.91 (5.79; 17.11–34.39)	4.00	6.00 (0.00)
London	29	10 (34)	24.52 (4.73; 20.00–36.00)	81	25 (31)	22.61 (4.41; 18.00–38.00)	6	0	23.99 (4.41; 18.00–28.92)	62	20 (32)	22.26 (4.62; 18.00–38.00)	13	5 (38)	23.67 (3.19; 19.00–31.00)	7.41	17.87 (8.07)
Maastricht	38	12 (32)	25.61 (5.68; 18.35–39.25)	48	14 (29)	20.19 (4.10; 12.00–29.00)	6	2 (33)	20.50 (5.17; 15.00–26.00)	25	8 (32)	18.64 (3.44; 12.00–27.00)	17	4 (24)	22.35 (3.81; 17.00–29.00)	12.50	NA
MHRC	51	0	22.21 (2.81; 16.07–27.07)	38	0	20.11 (2.50; 16.30–27.59)	3	0	19.02 (2.37; 16.30–20.60)	35	0	20.20 (2.52; 16.72–27.59)	0	NA	NA	7.89	30.18 (6.64)
MPRC	20	8 (40)	17.75 (4.31; 12.00–24.00)	30	15 (50)	17.13 (3.25; 12.00–22.00)	3	1 (33)	16.00 (4.36; 13.00–21.00)	9	4 (44)	16.00 (2.78; 12.00–20.00)	18	10 (56)	17.89 (3.25; 12.00–22.00)	10.00	7.32 (1.17)
Oslo Region	62	23 (37)	19.90 (3.62; 15.20–29.40)	21	8 (38)	19.88 (3.63; 15.55–29.08)	2	1 (50)	20.01 (5.43; 16.18–23.85)	18	7 (39)	19.85 (3.71; 15.55–29.08)	1	0	20.13	9.52	12.50 (1.40)
Pitt	64	26 (41)	22.70 (5.56; 14.03–38.24)	26	14 (54)	20.75 (5.32; 12.39–35.84)	2	1 (50)	17.05 (0.72; 16.55–17.56)	11	7 (64)	19.52 (6.38; 12.39–35.84)	13	6 (46)	22.36 (4.30; 16.07–30.66)	7.69	13.09 (3.62)
Singapore	53	25 (47)	21.96 (4.18; 14.51–29.84)	100	32 (32)	21.92 (3.57; 14.52–29.77)	11	3 (27)	20.08 (3.06; 14.76–26.50)	88	28 (32)	22.14 (3.60; 14.52–29.77)	1	1 (100)	22.64	11.00	18.98 (5.92)
SNUH	74	24 (32)	21.23 (2.49; 17.00–27.00)	74	19 (26)	20.70 (3.77; 15.00–34.00)	9	3 (33)	22.00 (4.97; 16.00–33.00)	46	11 (24)	20.37 (3.70; 15.00–34.00)	19	5 (26)	20.89 (3.35; 15.00–25.00)	12.16	26.71 (17.42)
Toho	16	8 (50)	23.19 (2.86; 18.00–28.00)	40	28 (70)	23.73 (6.95; 13.00–39.00)	4	3 (75)	19.00 (4.40; 14.00–24.00)	36	25 (69)	24.25 (7.02; 13.00–39.00)	0	NA	NA	10.00	12.00 (0.00)
Tokyo	25	12 (48)	22.08 (2.84; 16.00–25.00)	39	18 (46)	20.92 (3.50; 14.00–29.00)	3	0	22.67 (4.73; 19.00–28.00)	25	15 (60)	20.48 (3.10; 14.00–27.00)	11	3 (27)	21.45 (4.18; 16.00–29.00)	7.69	19.33 (15.49)
Toronto	39	16 (41)	25.46 (5.21; 18.17–38.24)	27	12 (44)	20.77 (1.84; 18.12–26.65)	0	NA	NA	4	2 (50)	19.70 (1.09; 18.12–20.55)	23	10 (43)	20.96 (1.90; 18.25–26.65)	NA	24.00 (0.00)
Toyama	141	67 (48)	25.07 (4.22; 18.00–38.00)	78	35 (45)	18.47 (4.06; 12.60–31.40)	10	3 (30)	20.76 (5.72; 14.90–31.40)	68	32 (47)	18.13 (3.69; 12.60–29.80)	0	NA	NA	12.82	36.79 (34.01)
UCSF	103	43 (42)	23.74 (7.55; 12.82–39.87)	70	33 (47)	19.39 (4.37; 12.39–32.36)	13	5 (38)	21.35 (4.40; 15.85–29.00)	46	22 (48)	19.04 (4.13; 12.39–28.75)	11	6 (55)	18.54 (5.08; 13.76–32.36)	18.57	19.64 (6.53)
Zurich	43	22 (51)	22.23 (5.56; 13.00–36.00)	60	25 (42)	19.20 (4.90; 13.00–35.00)	11	2 (18)	18.55 (3.36; 14.00–24.00)	34	14 (41)	19.15 (5.43; 13.00–35.00)	15	9 (60)	19.80 (4.78; 15.00–33.00)	18.33	15.48 (14.05)

*HC* healthy control, *CHR* clinical high risk for psychosis, *CHR-PS+* individuals at CHR who developed psychosis later individuals at CHR who developed psychosis later, *CHR-PS-* individuals at CHR who did not develop psychosis later, *CHR-UNK* could not follow up, *SD* standard deviation. Site name abbreviations as follows: Columbia, New York State Psychiatric Institute, Columbia University, New York; Copenhagen, Mental Health Center Copenhagen and CINS, Mental Health Center Glostrup, University of Copenhagen, Copenhagen, Denmark; CSU, Central South University, Changsha, China; Glasgow, Institute of Neuroscience and Psychology, University of Glasgow, Glasgow, Scotland; Heidelberg, Heidelberg University Hospital, Heidelberg, Germany; IDIBAPS, August Pi I Sunyer Biomedical Research Institute, Barcelona, Spain; ISMMS, Icahn School of Medicine at Mount Sinai, New York, New York; London, Institute of Psychiatry, Psychology and Neuroscience, King’s College London, London, United Kingdom; Maastricht, Maastricht University, Maastricht, the Netherlands; MHRC, Mental Health Research Center Moscow, Moscow, Russia; MPRC, Maryland Psychiatric Research Center, University of Maryland School of Medicine, Baltimore; Oslo region, NORMENT, University of Oslo and Oslo University Hospital, Oslo, Norway; Pitt, University of Pittsburgh, Pittsburgh, Pennsylvania; Singapore, Institute of Mental Health and National University of Singapore, Singapore; SNUH, Seoul National University, Seoul, Republic of Korea; Toho, Department of Neuropsychiatry, Toho University School of Medicine, Tokyo, Japan; Tokyo, Department of Neuropsychiatry, Graduate School of Medicine, The University of Tokyo, Tokyo, Japan; Toronto, Centre for Addiction and Mental Health, University of Toronto, Toronto, Ontario, Canada; Toyama, University of Toyama Graduate School of Medicine and Pharmaceutical Sciences, Toyama, Japan; UCSF, University of California, San Francisco; Zurich, Psychiatric Hospital, University of Zurich, Zurich, Switzerland. Additional site details can be found in the Supplement.

**Fig. 1:**
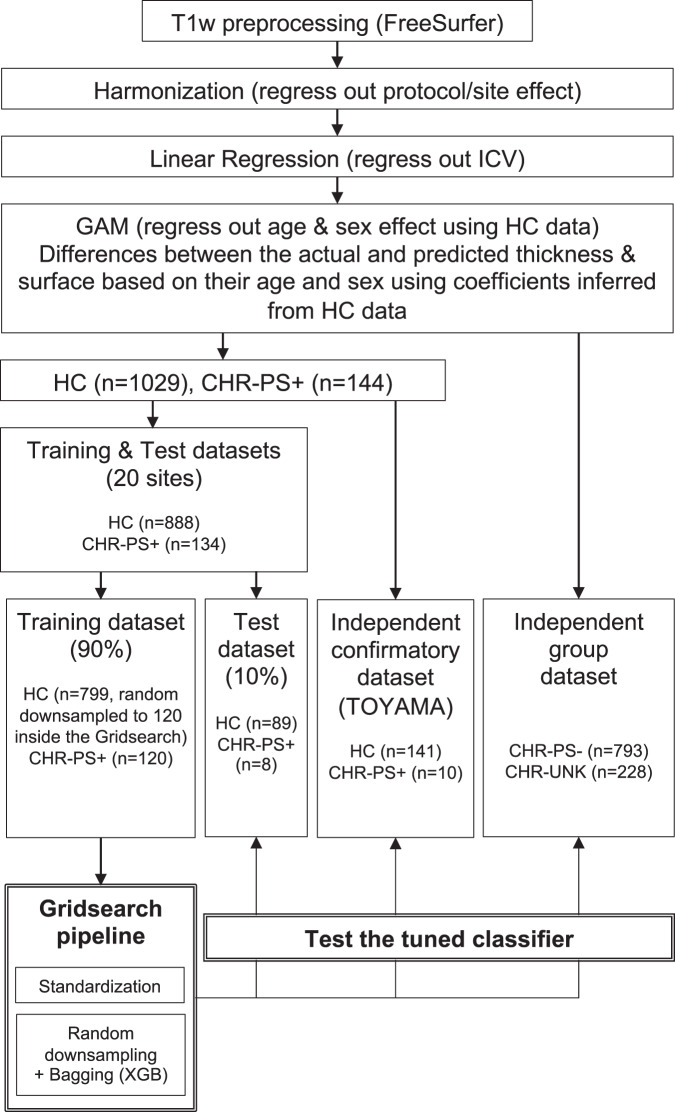
Diagram employed for the processing and analysis. HC healthy control, CHR clinical high risk for psychosis, CHR-PS+ individuals at CHR who developed psychosis later, CHR-PS- individuals at CHR who did not develop psychosis later, CHR-UNK individuals at CHR who could not follow up, SD standard deviation.

We applied a two-step approach [49] to evaluate the performance of the models by dividing the data into four datasets: training, test, independent confirmatory, and independent group datasets (Fig. 1). Test and independent confirmatory datasets were used as external validation datasets. First, the training and test datasets comprised the data from CHR-PS+ and HC from 20 sites except for Toyama, which was used as the independent confirmatory dataset. We chose this dataset because the Toyama site contributed the largest HC sample and excluding this dataset reduced sample imbalance between groups in building a machine learning classifier. Ninety percent of the data were randomly sorted as the training dataset, and the remaining 10% as the test dataset. A Kolmogorov–Smirnov test did not show any significant differences between training and test datasets in any structural features. The independent confirmatory dataset comprised the data from HCs and CHRs at the Toyama site; this data was completely excluded from the training partition, and was used to perform an independent first-step evaluation without site information leakage. To evaluate the classifier on unseen new data, we defined the CHR-PS− and CHR-UNK individuals in all sites as the independent group dataset to perform the second step.

### MRI data acquisition and preprocessing

#### Image acquisition and Processing

Participating sites contributed to T1-weighted MRI brain scans from 31 MRI scanners, including 29 3-T scanners and 2 1.5-T scanners (Supplementary Table S2). Detailed scan protocols and the number of scans for each protocol are described in the Supplementary Materials. After processing the data using FreeSurfer analysis software at each site [19–21], we extracted structural features from 153 regions of interest (ROI) including 68 regional measures of cortical thickness, 68 surface area (SA), 16 subcortical volume, and one intracranial volume according to the Desikan-Killiany atlas [69]. We implemented the ENIGMA consortium quality assessment pipeline [40, 41, 70–73] and 8 samples were excluded for lacking 20% of the ROIs data. Remaining missing values (1.20%) were imputed using a *k*-Nearest Neighbor (*k* = 3) approach.

### ComBat harmonization

ComBat [57] is a harmonization method used to remove scanner and protocol effects based on an adjusted general linear model harmonization method. Based on recent work demonstrating that neuroComBat harmonization increases statistical power within a mega-analytic framework, primary analyses were conducted within a mega-analysis framework using data that were corrected for site and scanner associations using neuroComBat harmonization [74]. Further analyses were conducted using Python version 3.7.12. We applied the extracted cortical thickness, surface area, subcortical volume, and intracranial volume measures with participants’ age and sex as covariates, along with protocol and site effects. To confirm that group status had no significant influence on the ComBat harmonization steps, we also conducted ComBat harmonization using the training dataset only (see Supplementary Materials).

### Features engineering

First, we fitted a general linear model to regress out effects of intracranial volume. Next, we fitted GAMs to only the HC data to estimate non-linear effects of age and sex for every structural feature; then we applied the fitted GAMs to obtain non-linear age- and sex-corrected features. To verify the absence of information leakage and the stability of the GAMs, we also repeated this procedure 1000 times on randomly sub-sampled HC data to estimate the GAMs (see Supplementary Materials and Fig. S1).

### XGBoost

XGBoost is a scalable tree boosting algorithm [62]. We applied standardization for the structural features to building a classifier. The use of input data standardization, optimization of the hyperparameters of the classifier (eta, min_child_weight, max_depth, subsample, colsample_bytree) were tuned using GridSearchCV implemented in the ‘scikit-learn’ module (version 1.0.2) in Python (https://scikit-learn.org/stable/auto_examples/release_highlights/plot_release_highlights_1_0_0.html) [75]. We plotted the weights of the classifier to determine the importance of the features for generalization. The classifier was optimized using a tenfold cross-validated grid search over a defined parameter grid. Data from the HC group were randomly downsampled to the same sample size as the CHR-PS+ group in each fold. To reduce downsampling bias, downsampling and grid search were repeated 1000 times and stratified tenfold for the training data. Then, we applied tenfold cross-validation and 1000 permutations to evaluate the significance of the cross-validation scores of the model with the best hyperparameters for the training dataset. The best cross-validation accuracy score was averaged across 1000 repeats. Permutation tests were conducted by shuffling the labels in the training data, and the permutation-based *p*-value was calculated [76]. The final model with the best hyperparameters was trained using the entire training dataset. Finally, the trained classifier was applied to the test set and the independent confirmatory dataset with the best parameters tuned by grid search. The predict probability was calculated by the trained classifier for each sample. Predict probability ranges from 0 to 1, with smaller values indicating more likely classification as CHR-PS+. The cut-off point for the predictive performance was set to 0.5.

The predictive performance of the classifier was evaluated using an independent group dataset (CHR-PS− and CHR-UNK). We compared the classifiers built from four different feature sets: (i) only cortical thickness values, (ii) only surface area values, (iii) only subcortical volumes only, and (iv) all features. The classifier with the best predictive performance for the independent confirmatory dataset was used for subsequent analysis.

### Statistical analysis

#### Evaluation metrics

First, the classifier was evaluated using the test, independent confirmatory, and independent group datasets by the given scores of the tuned classifier. We calculated the confusion matrix, macro, and weighted average accuracies to evaluate the classifier because the data used were imbalanced (see Supplementary Materials) [49].

#### Predictive performance of the classifier

The predictive performance of the classifier was defined as its performance on unseen data (in the independent confirmatory/group datasets) and was assessed using standard evaluation metrics. Chi-squared tests were applied to the classified labels of the test, independent confirmatory, and independent group datasets. Since we conducted a total of 6 comparisons, a Bonferroni’s correction was applied to adjust for the multiple statistical comparisons (*p* < 0.05/6 = 0.008). Predict probabilities generated by the XGBoost classifier were also tested using a nonparametric analysis of variance for all samples. To confirm little difference in the predictive performance between the assessments of CHR state, we tested the difference of predict probabilities including SIPS or CAARMS as a covariate. We also tested the difference in the rates of individuals predicted as CHR-PS+ that were assessed by either SIPS or CAARMS using a Chi-squared test. A GAM was used to assess non-linear relationships between age and the predictive performance of the classifier. Moreover, we conducted 4 comparisons (HCs vs. CHR-PS+, CHR-PS+ vs. CHR-PS-, CHR-PS+ vs. CHR-UNK and CHR-PS+ vs. CHR-PS- and CHR-UNK) of the decision curve analysis [77–79] using ‘dcurves’ package (version 0.4.0) in R software to estimate the classifier as well. Net benefit was calculated across a range of threshold probabilities [80] in comparison to getting MRI measurements to get a prediction for all patients or no patients. As threshold probabilities were set up to 50% (i.e., chance level), net benefit = sensitivity × prevalence – (1 – specificity) × (1 – prevalence) × 50%.

#### Relationship between predict probability and demographic and clinical characteristics

We tested the difference in the predictive performance with respect to sex and the existence of APSS, BLIPS, and GRDS using *t* tests (*p* < 0.05/3 = 0.016). Pearson’s correlation analyses were also conducted between standardized IQ and the predict probability. Bonferroni’s correction was applied to the subscores (*p* < 0.05/4 = 0.0125). To determine the relationship between the predict probability and symptom severity, Pearson’s correlation analyses were performed using the SIPS and CAARMS subscores for CHR-PS+, CHR-PS-, and CHR-UNK groups. We tested *z*-score normalized positive, negative and general subscores of the SIPS and CAARMS using Pearson’s correlation coefficients. Bonferroni’s correction was applied to the SIPS or CAARMS subscores (for SIPS: positive, negative, disorganization and general symptoms, *p* < 0.05/4 = 0.0125; for CAARMS: positive symptoms, cognitive change, emotional disturbance, negative symptoms, behavioral change, motor/physical changes, *p* < 0.05/6 = 0.0083). To determine the potential effect of antipsychotic medication on the classification, we also tested the difference in predict probabilities between those with and without medication use for each CHR subgroup using a *t*-test.

## Results

### Model evaluation

**Fig. 2:**
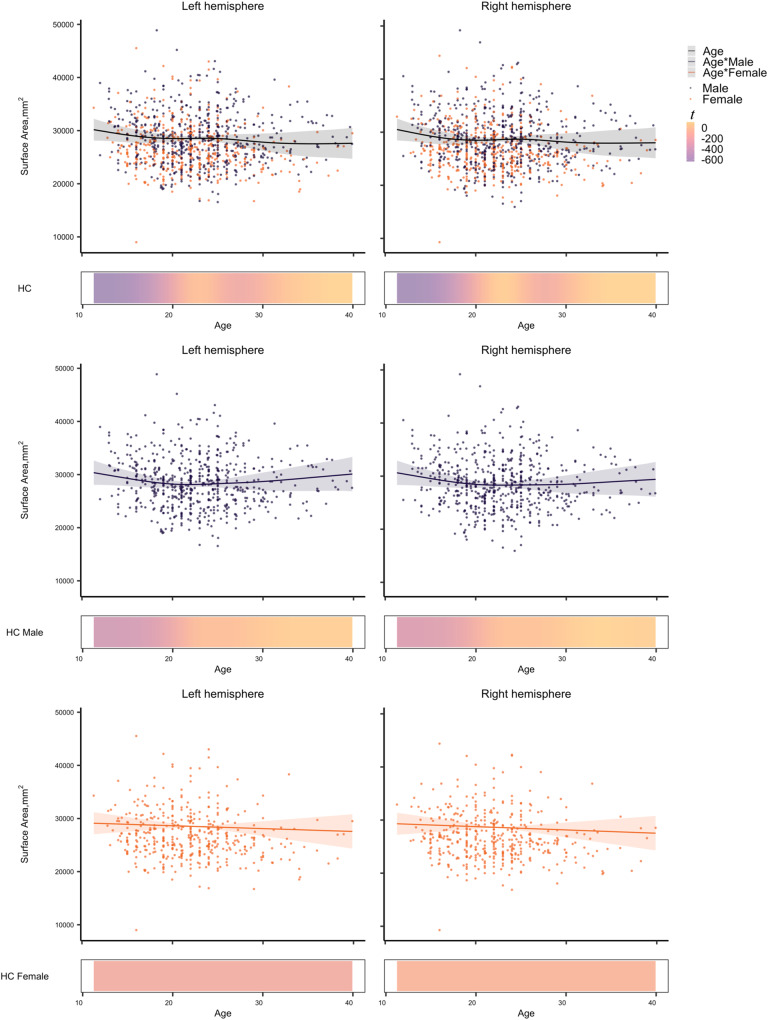
Non-linear age associations of the surface area in healthy controls. Each graph shows a partial effect of the best fit in GAMs. Shading around the line indicates the standard error. The bar underneath the age plots reflects the derivative of the slope.

**Fig. 3:**
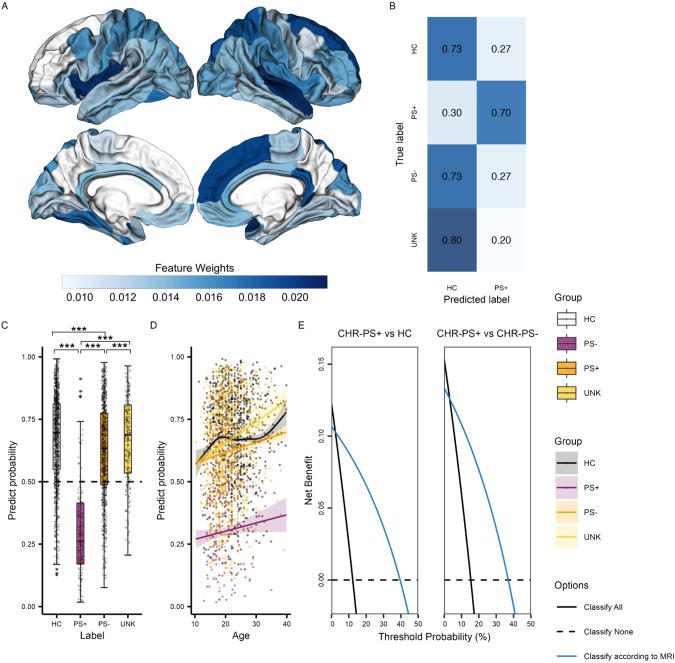
Surface area feature contributions and predictive performance comparisons of the XGBoost classifier. **A** Weighted surface area features of XGBoost classification in Desikan-Killiany atlas. **B** Predictive performance of HC and CHR-PS+ groups was evaluated using the independent confirmatory dataset, and CHR-PS- and CHR-UNK groups using the independent group dataset. **C** Box and scatter plot of predict probabilities of XGBoost. *P*-values of post hoc comparisons were corrected using a Bonferroni method (***p < 0.001, **p < 0.01, *p < 0.05). **D** Best fit for the association of age with the predict probability in a GAM. Shading around the line indicates the standard error. **E** Decision curve analysis showed the benefits of XGBoost predicting the risk of psychosis conversion according to MRI scan.

**Fig. 4:**
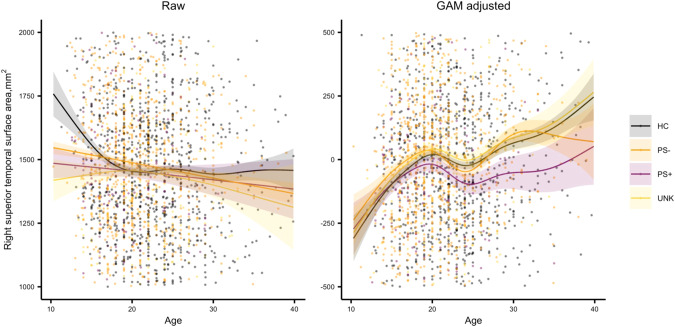
Age association of the surface area in the right superior temporal gyrus. Each graph shows a GAM fit of age, group, and age by group interaction. Shading around the line indicates the standard error.

A non-linear effect of age, sex, and age x sex interaction on SA was found in HCs, as shown in Fig. 2. The classifier using only non-linear fitted SA features (i.e., fit to HCs, applied to all) obtained the best performance in differentiating HC and CHR-PS+ groups (Supplementary Table S3). For the SA model, the best cross-validation accuracy within the training dataset was 85% (± 0.00008). The permutation test showed that the classifier performed significantly better than chance level (50%, *p* < 0.001). The accuracies with the best estimator for the test and independent confirmatory datasets were 68% and 73% (Fig. 3B), respectively. Regions with the top ten largest features weights were the superior temporal, insula, superior frontal, superior parietal, fusiform, isthmus of cingulate, parahippocampal gyri, and postcentral gyri to differentiate HC from CHR-PS+ groups (Fig. 3A, Supplementary Table S3). For SA in the right superior temporal gyrus, which was the strongest contributing feature of the classifier, the ComBat harmonized feature showed no significant difference among the groups (*p* > 0.05), while ComBat harmonized and non-linear age- and sex-adjusted feature revealed a difference between CHR-PS+ and CHR-PS- (*t* = 2.137, *p* = 0.0327), and CHR-PS+ and CHR-UNK (*t* = 2.140, *p* = 0.0325; Fig. 4).

For a confirmatory analysis, machine learning classifiers using 152 sMRI raw brain characteristics showed poorer performance compared to the corresponding age- and sex-adjusted machine learning classifiers (Supplementary Materials). We also tried to build classifiers to differentiate CHR from HCs or CHR-PS+ from CHR-PS-, however, those ones only showed approximate chance level (50%) accuracies.

### Predictive performance of the classifier for the test, independent confirmatory, and independent group datasets

A chi-squared test showed a significant difference in the classified labels for the independent confirmatory, and independent group datasets, respectively (*X*^2^(1, *n* = 151) = 6.34, *p* = 0.012 and *X*^2^(1, *n* = 1021) = 4.39, *p* = 0.036). Further residual analysis showed that the HC group was significantly more likely to be classified as HCs than the CHR-PS+ group (73% vs. 30%, *corrected p* = 0.004, Fig. 3B). For the independent group dataset, no difference between CHR-PS- and CHR-UNK groups was found (73% vs. 80%, *corrected p* = 0.029).

For the overall sample, a chi-square test showed a significant difference in the classified labels between the four groups (*X*^2^(3, 1172) = 15.12, *p* = 0.002). Further residual analysis showed a significant difference in the classified labels between CHR-PS+ and the other three groups (*Bonferroni corrected p’s* < 0.05; Fig. 3B). For the predict probability, an Kruskal-Wallis test showed a significant difference between the four groups (*H* = *278.86*, *p* < 0.001). Post-hoc comparisons showed that CHR-PS+ group was different from all other groups and that the CHR-PS- group was in between CHR-PS+ and HC groups (HC > CHR-PS- > CHR-PS+), while the predict probability did not differ between CHR-UNK and HCs (CHR-UNK > CHR-PS- > CHR-PS+; *Bonferroni corrected p*’s < 0.05; Fig. 3C). The difference changed little after controlling the methods of the CHR assessments as a covariate (CHR group: *F*(2, 1161) = 192.25, *p* < 0.001; Assessment method: *F*(1, 1161) = 0.00, *p* > 0.05), and CHR individuals predicted as CHR-PS+ did not differ between participants assessed with SIPS versus CAARMS, *X*^2^(1, 1449) = 2.59, *p* > 0.05; (Supplementary Table S4). Although the classifier was built according to the features after controlling for non-linear age effect, a GAM analysis demonstrated that the predict probability was associated with age (*F* = 11.33, *p* = 0.003), and differed between CHR-PS+ and HC (*t* = 20.72, *p* < 0.001), CHR-PS+ and CHR-PS- (*t* = 17.83, *p* < 0.001), and CHR-PS+ and CHR-UNK (*t* = 17.64, *p* < 0.001; Fig. 3D). No significant age × group interaction was found in the predict probability.

The estimated decision curve for all comparisons (HCs vs. CHR-PS+, CHR-PS+ vs. CHR-PS-, CHR-PS+ vs. CHR-UNK and CHR-PS+ vs. CHR-PS- and CHR-UNK) showed that in clinical setting, compared to MRI measurement for all patients or no MRIs at all, getting a prediction from current classifier/model leads to higher net benefit to discoverer transition of CHR (Fig. 3E).

### Relationship between predict probability and demographic and clinical characteristics

We observed no effects of sex or APSS, BLIPS, or GRDS status, on the predict probability (*p* > 0.05). No significant correlations were found between standardized IQ and the predict probability for each group. No significant correlation was found between symptom severity scores and predict probability. No significant difference was found for the antipsychotics use was found among each CHR group (*p* > 0.05).

## Discussion

To the best of our knowledge, the current study is the one of a few to apply a machine learning approach to discriminate HC and CHR-PS+ groups in a large multisite sample [12]. To evaluate the classifier, we employed a two-step approach using an independent confirmatory dataset, obtained at a different site and using a different protocol from the ones used to build the classifier; we also used an independent group dataset including CHR-PS- and CHR-UNK groups. Although previous study reported 94% accuracy [12], we have achieved 85% accuracy in the 2-class classification in the training dataset using non-linear adjustment of SA features for age and sex. The patterns of neuroanatomical alterations were also useful in identifying CHR-PS- individuals. Specially, of the CHR groups, the CHR-UNK group was the most likely to be classified as HC by the classifier, than those in other CHR groups, showing no difference in the predict probability from HC.

In this study, we were able to differentiate HC from CHR-PS+ group with 85% and 68% accuracy in the training and test sets, respectively. The performance accuracy achieved by the classifier on the independent confirmatory dataset was 73%. In contrast to prior studies [12, 38, 53], we successfully built a model with promising predictive performance for new data. Our findings suggest that ComBat is not only useful to increase statistical power [55, 57, 74] but also crucial for improving the accuracy in building a machine learning model out of multisite data. As expected, the majority of CHR-PS- and CHR-UNK individuals were classified as HCs. Moreover, no significant associations were found between the predict probability and sex or IQ, or antipsychotics use for each CHR group. We suggest that a machine learning classifier trained to identify differences between CHR-PS+ and healthy controls may be helpful to identify UHR individuals at risk for conversion.

In line with prior studies of cortical alterations in CHR [11, 37–39, 43], we found that the pattern of SA features, including the superior temporal, insula, superior frontal, superior parietal, fusiform, isthmus of cingulate, and parahippocampal gyri, contributed to identifying CHR-PS+ from HCs (Fig. 2A, Fig. 4). These findings align with previous work reporting (right) superior temporal gyrus functional alterations may underlie deficits in (non-)emotional multisensory integration in schizophrenia patients [81] and working memory-related dysfunction in CHR [82]. CHR individuals who converted or presented with greater clinical symptom within a 2-year follow-up exhibited smaller SA in the rostral anterior cingulate, lateral and medial prefrontal regions, and parahippocampal gyrus [11]. SA is more closely related to volume than cortical thickness [24], and the volume of the isthmus of cingulate gyrus has been reported to be different in resilient and non-resilient CHR individuals [39]. The neuroanatomical alteration/ deviance pattern of SA found in the current study between HCs and CHR-PS+ groups are consistent with findings from other studies, which implicate the volume of superior temporal, frontal and fusiform regions in CHR transitions [27, 38] and schizophrenia [29, 41, 42]. Our initial ENIGMA CHR study showed the differences mainly in CT, and for two regions (i.e., the paracentral lobule and fusiform gyrus), the non-linear pattern of the age trajectory differed between HC and CHR [27, 83]. However, the former study was focused on the statistical significance, and the current study is focused on predictive performance. As traditional significance approaches do not capture predictive variable sets [84], resulting in SA serving better building a predictive model. It is possible that the current study engineered the features that made the differences in SA more prominent, by using GAM to estimate the brain age gap in a non-linear manner. Moreover, as the result of GAM eliminating the non-linear adolescent development of SA in differentiating HCs and CHR-PS+, our classifier achieved promising generalization of predictive performance.

Although we did not find any difference in predict probability between APSS, BLIPS, or GRDS status, it is important to note that previous studies demonstrated CHR subgroup-specific changes in sMRI metrics [85], such as subcortical volume reductions in left anterior frontal, right caudate, right hippocampus, and amygdala in CHR with a genetic risk, while CHR with attenuated psychotic symptoms exhibited right middle temporal cortical reduction [86]. Moreover, studies have shown that transition rates may differ between CHR subgroups [87]. These findings underscore the importance of using adequate sampling of CHR participants across subgroups and different clinical stages. Such efforts may result in more accurate predictive models in the future.

The predict probability given by the classifier based on the neuroanatomical deviance showed significant differences among the HC or CHR-UNK, CHR-PS-, and CHR-PS+ groups at baseline (HC, CHR-UNK > CHR-PS- > CHR-PS+; Fig. 3C). The results suggest that predict probability is a useful index allowing us to better understand how neuroanatomical deviance is associated with psychosis conversion. This further implies that the neuroanatomical deviance was already observed at baseline in CHR-PS+ group. Moreover, in contrast to previous working reporting a positive association between age conversion rates [83], our observed association between predict probability and age (Fig. 3D) could suggest that the likelihood of a HC prediction increases with age. One possibility for this finding is the distribution of age across groups. Specifically, participants older than 30 years old were sparsely distributed in all groups in the current study, which may result in spurious associations between age and predict probability. To understand the exact nature of the association between age and predict probability, more data of CHR participants of older ages is necessary. These results suggest that psychosis-related brain characteristics may decrease according to brain development which may effect on the onset of psychosis.

Our study has several limitations. First, to harmonize site effects, ComBat was applied to both HC and CHR subjects which by assuming a common covariate model (that is typically preserved by ComBat) might potentially lead to an information leak [88]. However, without traveling subject harmonization, ComBat was considered the most appropriate method for testing a classifier on individual samples from multi-site datasets [41–43]. Second, we could not test the effect of psychosis-by-age interaction on the predict probability as longitudinal MRI data were not available. Longitudinally tracking neuroanatomical changes around the onset of psychosis would offer more detailed information to understand the progressive brain pathology. Third, substance use of cannabis or alcohol was not available for the current study which is reported associated with increased risk of developing depression in young adulthood [89]. Fourth, while we note that a classifier that can distinguish between CHR-PS+ and CHR-PS- status is clinically useful, we did not explicitly train our classifier to distinguish between CHR-PS+ and CHR-PS-. Previous work suggests that the magnitude of differences in MRI metrics between CHR-PS+ and CHR-PS- are small. Although there exist no well-validated methods to decide on the minimal sample size to create a reliable classifier, considering these subtle differences, the sample size of CHR-PS+ may likely be insufficient. Increased availability of CHR data may enable the development of such a classifier in the future.

In conclusion, we successfully trained a 2-class XGBoost classifier (HC versus CHR-PS+) and showed promising predictive performance on a multi-site dataset after considering age and sex differences. This classifier successfully identified 73% of CHR-PS- individuals as HC, and further 80% of CHR individuals who were not follow-up for the onset. These results suggest that when considering adolescent brain development, baseline MRI scans for CHR individuals may be helpful to identify their prognosis. Especially, the superior temporal, insula, superior frontal areas contributed most in differentiating CHR-PS+ from HCs. In light previous work reporting that alterations in these regions have implicated in psychosis onset, these areas could be informative in improving understanding of pathophysiology linked to psychosis onset. Future prospective studies are required about what and how the psychosis-related brain characteristics change according to the adolescent development, and whether the classifier could be helpful in the clinical settings.

### Supplementary information

supplemental material

Supplementary Figure S1

## Data Availability

The Python code used to build the classifier is openly available on GitHub: https://github.com/yh-zhu/MolPsy_2024_ENIGMA.git.
