# Supplementary material for: Using brain structural neuroimaging measures to predict psychosis onset for individuals at clinical high-risk
Source: Mol Psychiatry. 2024 Feb 9;29(5):1465–77. doi: 10.1038/s41380-024-02426-7 (PMC11189817; doi:10.1038/s41380-024-02426-7)
Supplement: Supplementary file 1 — supplemental material [file 41380_2024_2426_MOESM1_ESM.docx]

**Using Structural Neuroimaging Measures to Predict Psychosis Onset for Individuals at Clinical High-Risk**

**Supplementary Materials**

**Contents**

[**Supplementary Methods** 2](#_Toc149607068)

[Confirmation of ComBat harmonization 2](#_Toc149607069)

[Verification of GAMs 2](#_Toc149607070)

[Evaluation metrics 2](#_Toc149607071)

[**Supplementary Results** 4](#_Toc149607072)

[Performance of classifier built only on cortical thickness features 4](#_Toc149607073)

[Performance of classifier built only on subcortical volume features 4](#_Toc149607074)

[Performance of classifier built on all sMRI features 4](#_Toc149607075)

[Performance of classifier built on all sMRI features without HC based non-linear age and sex adjustment 5](#_Toc149607076)

[**Supplementary Figures** 6](#_Toc149607077)

[Supplementary Figure S1. Visualization of GAMs. Distributions showed randomly sub-sampled 70% of the HC participants 1000 times coefficients’ p-values in top 10 features contributed to XGBoost classification. The coefficients’ p-values of fitted GAMs using all HC data showed in red dashed line. 6](#_Toc149607078)

[**Supplementary Tables** 7](#_Toc149607079)

[Supplementary Table S1. Site information. 7](#_Toc149607080)

[Supplementary Table S2. Scanner and acquisition parameters in the participating sites. 8](#_Toc149607081)

[Supplementary Table S3. Feature weights contributed to XGBoost classification. 9](#_Toc149607082)

[Supplementary Table S4. Proportions of CHR-PS+ individuals diagnosed by SIIPS or CAARMS predicted as CHR-PS+ 11](#_Toc149607083)

# **Supplementary Methods**

## **Confirmation of ComBat harmonization**

We conducted ComBat harmonization using the training dataset only. As there was only one HC participant from MPRC (Table S1), data acquired from this site was excluded from further analysis. One-way random, single score of intraclass correlation coefficient (ICC) test was applied to harmonized cortical thickness, SA, subcortical volume, and intracranial volume measures using all data and the training dataset only. The median of coefficients was 0.98 (range 0.96-0.99). This indicated that there was little concern about potential information leak age in applying ComBat to the entire dataset.

## **Verification of GAMs**

We randomly sub-sampled 70% of the HC participants 1000 times and compared the coefficients’ p-values of fitted GAMs using all HC data (**Figure S1**).

## **Evaluation metrics**

We calculated the confusion matrix, macro, and weighted average accuracies to evaluate the classifier in view of imbalanced data as follows:

Considering,

$Precision =\frac{tp}{tp+fp}$,

$Recall=\frac{tp}{tp+fn}$,

$F1=\left( \frac{2}{{recall}^{-1}+{precision}^{-1}} \right)$, and

$Accuracy=\frac{tp+tn}{tp+tn+fp+fn}$,

where *tp*, true positive; *tn*, true negative; *fp*, false positive; and *fn*, false negative.

Precision, also referred to as the positive predictive value in statistical analysis, is defined as the ratio *tp* / (*tp* + *fp*), where the best value is 1 and the worst value is 0. Recall, also known as sensitivity, is the repletion of the type-II error rate. The F1-score is a weighted average of the precision and recall; the best value is 1 and the worst value is 0. We calculated the metrics for each group label to determine their unweighted mean as the macro average accuracy. The macro average does not consider label imbalance. For the weighted average accuracy, we calculated metrics for each group label and found their average weighted by the number of true instances for each group label. In this sense, the weighted average represents a variant of the ‘macro’ adjusted for group imbalance.

# **Supplementary Results**

## **Performance of classifier built only on cortical thickness features**

For the cortical thickness model the best cross-validation accuracy within the training dataset was 86% (±0.01). The accuracy with the best estimator for the test and independent confirmatory datasets were 70% and 80%, respectively. Although the accuracy scores seemed high, the predictive performance was low, as 93% and 70% of the true CHR-PS+ subjects of group from the test and independent confirmatory dataset were predicted as HCs.

## **Performance of classifier built only on subcortical volume features**

For this model, the best cross-validation accuracy within the training dataset was 81% (±0.02). The accuracy of the best estimator for the test and independent confirmatory datasets was 70% and 72%, respectively. Although these accuracy scores are high, the actual predictive performance was low, as 70% and 80% of the true CHR-PS+ subjects of the test and validation dataset were predicted as HCs.

## **Performance of classifier built on all sMRI features**

For the model using all 152 sMRI features, the best cross-validation accuracy within the training dataset was 87% (±0.01). The accuracy using the best estimator for the test and independent confirmatory datasets were 70% and 79%, respectively. Again, the actual predictive performance was low, as 93% and 70% of the CHR-PS+ subjects of the test and validation datasets were predicted as HCs.

## **Performance of classifier built on all sMRI features without HC based non-linear age and sex adjustment**

Here, the best cross-validation accuracy within the training dataset was 85% (±0.00008). The accuracy with the best estimator for the test and validation dataset was 68% and 73%, respectively.

# **Supplementary Figures**

## **Supplementary Figure S1. Visualization of GAMs.** Distributions showed randomly sub-sampled 70% of the HC participants 1000 times coefficients’ p-values in top 10 features contributed to XGBoost classification. The coefficients’ p-values of fitted GAMs using all HC data showed in red dashed line.

# **Supplementary Tables**

## **Supplementary Table S1. Site information.**

| Site | Scanner Manufacturer | HC | CHR | CHR-PS+ | CHR-PS- | CHR-UNK | Antipsychotic Use (n, %) | IQ Method | CHR Inclusion Criteria |
| --- | --- | --- | --- | --- | --- | --- | --- | --- | --- |
| Columbia | GE | 9 | 17 | 3 | 14 | 0 | 7 (41.18) | NA | SIPS |
| Copenhagen | Philips | 0 | 52 | 4 | 34 | 14 | 9 (17.31) | WAIS-III | CAARMS |
|  | Philips | 58 | 111 | 9 | 61 | 41 | 33 (29.73) | WAIS-III | CAARMS |
| CSU | Siemens | 59 | 52 | 21 | 31 | 0 | 0 (0.00) | WISC-I+PC | SIPS |
| Glasgow | Siemens | 46 | 80 | 6 | 74 | 0 | 2 (2.50) | NART | CAARMS |
| Heidelberg | Siemens | 24 | 8 | 0 | 5 | 3 | 0 (0.00) | WISC-IV | SIPS |
|  | Siemens | 9 | 14 | 0 | 13 | 1 | 0 (0.00) | WISC-IV | SIPS |
| IDIBAPS | Siemens | 39 | 51 | 13 | 30 | 8 | 28 (54.90) | WAIS-III, WISC-IV | SIPS |
|  | Siemens | 15 | 15 | 4 | 5 | 6 | 4 (26.67) | WAIS-III, WAIS-IV,  WISC-IV, WISC-V | SIPS |
|  | Siemens | 0 | 8 | 0 | 7 | 1 | 2 (25.00) | WAIS-IV, WISC-IV | CAARMS |
| ISMMS | Siemens | 12 | 25 | 1 | 12 | 12 | 6 (24.00) | NA | SIPS |
| London | GE | 29 | 81 | 6 | 62 | 13 | 8 (9.88) | NA | CAARMS |
| Maastricht | Philips | 33 | 48 | 6 | 25 | 17 | 9 (18.75) | DART | SIPS |
|  | Philips | 5 | 0 | 0 | 0 | 0 | 0 (0.00) | DART | SIPS |
| MHRC | Philips | 51 | 38 | 3 | 35 | 0 | 34 (89.47) | NA | SIPS |
| MPRC | Siemens | 19 | 10 | 3 | 5 | 2 | 2 (20.00) | WASI, WASI-II | SIPS |
|  | Siemens | 1 | 20 | 0 | 4 | 16 | 4 (20.00) | WASI-II | SIPS |
| Oslo Region | GE | 39 | 13 | 1 | 11 | 1 | 2 (15.38) | WASI | SIPS |
|  | GE | 23 | 8 | 1 | 7 | 0 | 2 (25.00) | WASI | SIPS |
| Pitt | Siemens | 64 | 26 | 2 | 11 | 13 | 9 (34.62) | WASI | SIPS |
| Singapore | Siemens | 53 | 100 | 11 | 88 | 1 | 1 (1.00) | NA | CAARMS |
| SNUH | Siemens | 74 | 74 | 9 | 46 | 19 | 14 (18.92) | K-WAIS | SIPS |
| Toho | Toshiba | 16 | 40 | 4 | 36 | 0 | 19 (47.50) | WAIS-III | SIPS |
| Tokyo | GE | 21 | 29 | 3 | 18 | 8 | 10 (34.48) | JART25 | SIPS |
|  | GE | 4 | 10 | 0 | 7 | 3 | 8 (80.00) | JART25 | SIPS |
| Toronto | GE | 39 | 27 | 0 | 4 | 23 | 6 (22.22) | RBANS | SIPS |
| Toyama | Siemens | 52 | 21 | 5 | 16 | 0 | 3 (14.29) | JART50 | CAARMS |
|  | Siemens | 89 | 57 | 5 | 52 | 0 | 12 (21.05) | JART50 | CAARMS |
| UCSF | Siemens | 103 | 70 | 13 | 46 | 11 | 13 (18.57) | WASI-II | SIPS |
| Zurich | Phillips | 5 | 26 | 3 | 19 | 4 | 7 (26.92) | MWT-B, WISC | SIPS |
|  | Phillips | 38 | 34 | 8 | 15 | 11 | 9 (26.47) | MWT-B, WISC | SIPS |

## **Supplementary Table S2. Scanner and acquisition parameters in the participating sites.**

| Site | Scanner Manufacturer | Scanner Model | Tesla Strength | Repetition Time (ms) | Echo Time (ms) | Flip Angle | Voxel Size (mm) | FreeSurfer version |
| --- | --- | --- | --- | --- | --- | --- | --- | --- |
| Columbia | GE | Discovery MR750 | 3 | 7840 | 3.1 | 12° | 0.8x0.8x0.8 | 6.0.0 |
| Copenhagen | Philips | Achieva | 3 | 10.028 | 4.6 | 8° | 0.75x0.75x0.80 | 6.0.0 |
|  | Philips | Achieva | 3 | 10.01 | 4.6 | 8° | 0.75x0.75x0.80 | 6.0.0 |
| CSU | Siemens | Skyra | 3 | 2530 | 2.33 | 7° | 1.0x1.0x1.0 | 6.0.0 |
| Glasgow | Siemens | Trio | 3 | 2250 | 2.6 | 9° | 1.0x1.0x1.0 | 6.0.0 |
| Heidelberg | Siemens | PET/MR | 3 | 2300 | 2.98 | 9° | 1.0x1.0x1.0 | 6.0.0 |
|  | Siemens | Tim Trio | 3 | 2300 | 2.98 | 9° | 1.0x1.0x1.0 | 6.0.0 |
| IDIBAPS | Siemens | Tim Trio | 3 | 2300 | 3.01 | 9° | 0.94x0.94x1.0 | 6.0.0 |
|  | Siemens | Prisma fit | 3 | 2300 | 3.01 | 9° | 0.94x0.94x1.0 | 6.0.0 |
|  | Siemens | Prisma fit | 3 | 2300 | 2.98 | 9° | 1.0x1.0x1.2 | 6.0.0 |
| ISMMS | Siemens | Skyra | 3 | 2400 | 2.07 | 8° | 0.8x0.8x0.8 | 6.0.0 |
| London | GE | Electric Discovery MR750 | 3 | 7.31 | 3.02 | 11° | 1.1x1.1.x1.2 | 6.0.0 |
| Maastricht | Philips | Intera | 3 | 2250 | 4.6 | 8° | 1.17×1.17×1.20 | 6.0.0 |
|  | Philips | Achieva | 3 | 2250 | 4.6 | 8° | 1.2x0.8x0.8 | 6.0.0 |
| MHRC | Philips | Achieva | 3 | 8.2 | 3.7 | 8° | 0.83×0.83x1.0 | 6.0.0 |
| MPRC | Siemens | Trio | 3 | 2400 | 2.2 | 8° | 0.8x0.8x0.8 | 6.0.0 |
|  | Siemens | Prisma | 3 | 2400 | 2.2 | 8° | 0.8x0.8x0.8 | 6.0.0 |
| Oslo Region | GE | Signa HDxt | 3 | 7800 | 2956 | 12° | 1.0×1.0×1.2 | 5.3 |
|  | GE | Discovery MR750 | 3 | 8,16 | 3,18 | 12° | 1.0×1.0×1.0 | 5.3 |
| Pitt | Siemens | Prisma | 3 | 2400 | 2.2 | 8° | 0.8x0.8x0.8 | 6.0.0 |
| Singapore | Siemens | Tim Trio | 3 | 2300 | 3 | 9° | 1.0x1.0x1.0 | 6.0.0 |
| SNUH | Siemens | Magnetom TrioTim | 3 | 1670 | 1.89 | 9° | 1x0.98x0.98 | 6.0.0 |
| Toho | Toshiba | EXCELART Vantage | 1.5 | 24.4 | 5.5 | 35° | 0.98×0.98×1.0 | 5.2 |
| Tokyo | GE | SIGNA HDx | 3 | 6.8 | 1.94 | 20° | 1.0x1.0x1.0 | 6.0.0 |
|  | GE | DISCOVERY MR750W | 3 | 8.46 | 3.25 | 20° | 1.0x1.0x1.0 | 6.0.0 |
| Toronto | GE | Discovery MR750 | 3 | 6736 | 2.99 | 8° | 0.9x0.9x0.9 | 6.0.0 |
| Toyama | Siemens | Magnetom Vision | 1.5 | 2400 | 5 | 40° | 1.0x1.0x1.0 | 6.0.0 |
|  | Siemens | Magnetom Verio | 3 | 2300 | 2.9 | 9° | 1.0x1.0x1.2 | 6.0.0 |
| UCSF | Siemens | Magnetom TrioTim | 3 | 2300 | 2.95 | 9° | 1.0x1.0x1.2 | 5.1 |
| Zurich | Phillips | Philips Achieva TX | 3 | 8.3 | 3.8 | 8° | 1.0x1.0x1.0 | 6.0.0 |
|  | Phillips | Philips Achieva TX | 3 | 8.3 | 3.8 | 8° | 1.0x1.0x1.0 | 6.0.0 |

## **Supplementary Table S3. Feature weights contributed to XGBoost classification.**

| **Regions** | **Weights** |
| --- | --- |
| R_superiortemporal_surfavg | 0.0214 |
| L_insula_surfavg | 0.0213 |
| R_superiorfrontal_surfavg | 0.0201 |
| R_superiorparietal_surfavg | 0.0192 |
| R_isthmuscingulate_surfavg | 0.0191 |
| L_fusiform_surfavg | 0.0187 |
| L_parahippocampal_surfavg | 0.0186 |
| L_frontalpole_surfavg | 0.0174 |
| R_insula_surfavg | 0.0171 |
| R_postcentral_surfavg | 0.0167 |
| R_transversetemporal_surfavg | 0.0165 |
| L_superiorparietal_surfavg | 0.0164 |
| R_supramarginal_surfavg | 0.0164 |
| R_cuneus_surfavg | 0.0163 |
| L_caudalmiddlefrontal_surfavg | 0.0162 |
| L_postcentral_surfavg | 0.0161 |
| R_lateralorbitofrontal_surfavg | 0.0159 |
| R_lateraloccipital_surfavg | 0.0157 |
| R_rostralmiddlefrontal_surfavg | 0.0157 |
| R_rostralanteriorcingulate_surfavg | 0.0157 |
| L_posteriorcingulate_surfavg | 0.0155 |
| L_rostralmiddlefrontal_surfavg | 0.0154 |
| R_bankssts_surfavg | 0.0152 |
| R_caudalanteriorcingulate_surfavg | 0.0149 |
| L_middletemporal_surfavg | 0.0149 |
| R_medialorbitofrontal_surfavg | 0.0149 |
| L_rostralanteriorcingulate_surfavg | 0.0149 |
| L_cuneus_surfavg | 0.0149 |
| L_superiorfrontal_surfavg | 0.0148 |
| R_precuneus_surfavg | 0.0148 |
| R_parsopercularis_surfavg | 0.0148 |
| L_caudalanteriorcingulate_surfavg | 0.0148 |
| L_isthmuscingulate_surfavg | 0.0147 |
| R_inferiorparietal_surfavg | 0.0147 |
| L_inferiortemporal_surfavg | 0.0146 |
| L_precentral_surfavg | 0.0146 |
| L_parstriangularis_surfavg | 0.0145 |
| L_lateraloccipital_surfavg | 0.0143 |
| L_inferiorparietal_surfavg | 0.0142 |
| R_temporalpole_surfavg | 0.0141 |
| L_lateralorbitofrontal_surfavg | 0.0139 |
| R_precentral_surfavg | 0.0139 |
| L_superiortemporal_surfavg | 0.0139 |
| R_middletemporal_surfavg | 0.0138 |
| R_paracentral_surfavg | 0.0136 |
| L_medialorbitofrontal_surfavg | 0.0135 |
| R_fusiform_surfavg | 0.0135 |
| L_precuneus_surfavg | 0.0134 |
| R_parstriangularis_surfavg | 0.0133 |
| L_temporalpole_surfavg | 0.0133 |
| L_parsopercularis_surfavg | 0.0132 |
| R_inferiortemporal_surfavg | 0.0131 |
| L_supramarginal_surfavg | 0.0131 |
| L_lingual_surfavg | 0.0127 |
| L_pericalcarine_surfavg | 0.0126 |
| R_frontalpole_surfavg | 0.0125 |
| L_entorhinal_surfavg | 0.0125 |
| R_parahippocampal_surfavg | 0.0125 |
| L_parsorbitalis_surfavg | 0.0124 |
| L_bankssts_surfavg | 0.0123 |
| R_lingual_surfavg | 0.0123 |
| R_posteriorcingulate_surfavg | 0.0123 |
| L_paracentral_surfavg | 0.0118 |
| R_entorhinal_surfavg | 0.0115 |
| R_caudalmiddlefrontal_surfavg | 0.0115 |
| R_parsorbitalis_surfavg | 0.0113 |
| L_transversetemporal_surfavg | 0.0110 |
| R_pericalcarine_surfavg | 0.0092 |

## **Supplementary Table S4. Proportions of CHR-PS+ individuals diagnosed by SIIPS or CAARMS predicted as CHR-PS+**

|  | **CHR-PS+** | **Predicted as CHR-PS+** | **Ratio** |
| --- | --- | --- | --- |
| SIPS | 98 | 84 | 86% |
| CARRMS | 46 | 41 | 89% |
