# Supplementary figures and images for: Using brain structural neuroimaging measures to predict psychosis onset for individuals at clinical high-risk
Source: Mol Psychiatry. 2024 Feb 9;29(5):1465–77. doi: 10.1038/s41380-024-02426-7 (PMC11189817; doi:10.1038/s41380-024-02426-7)

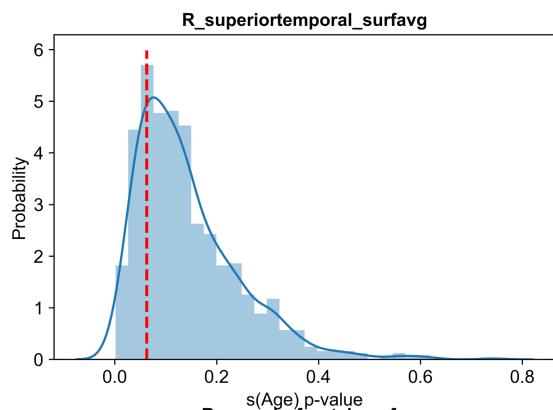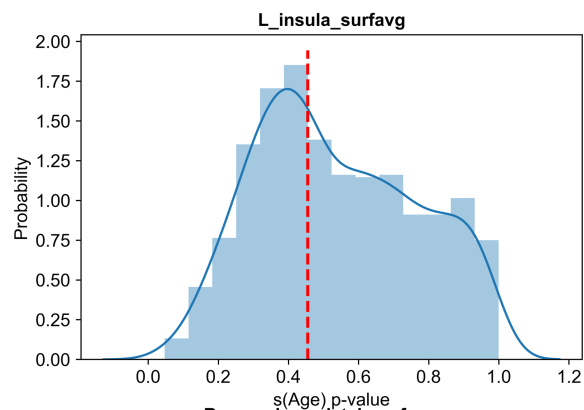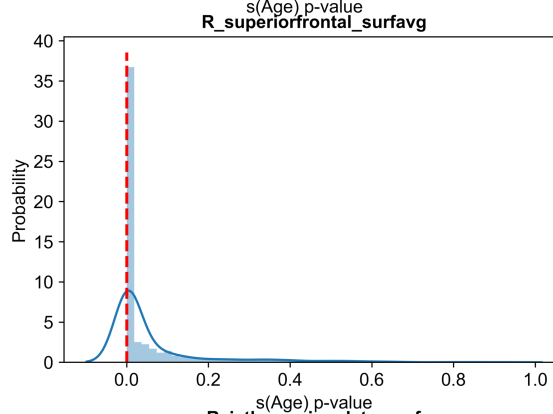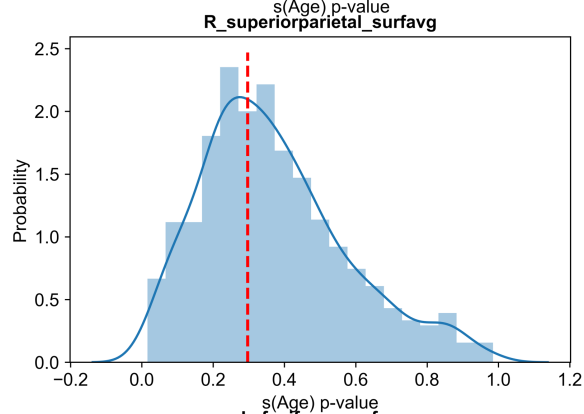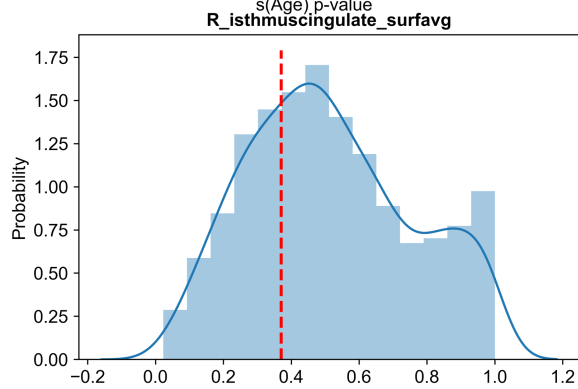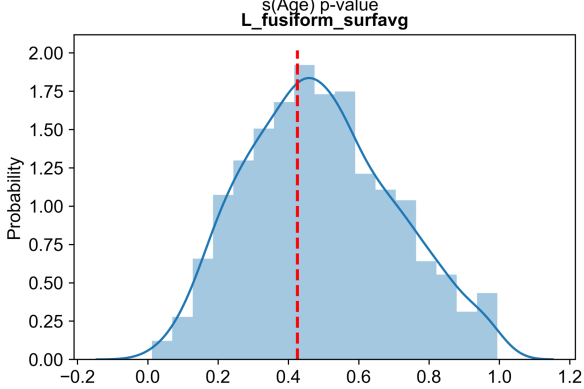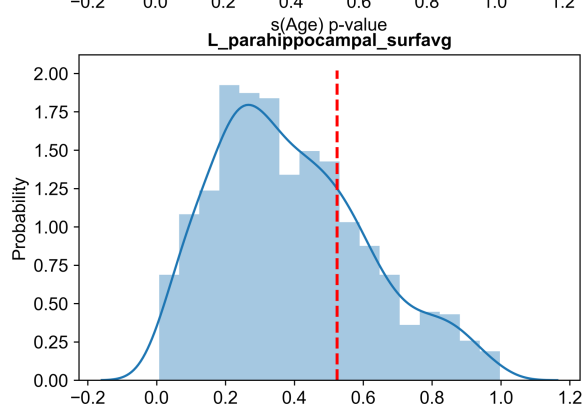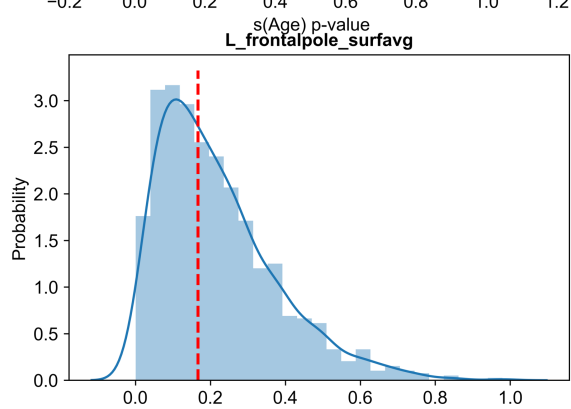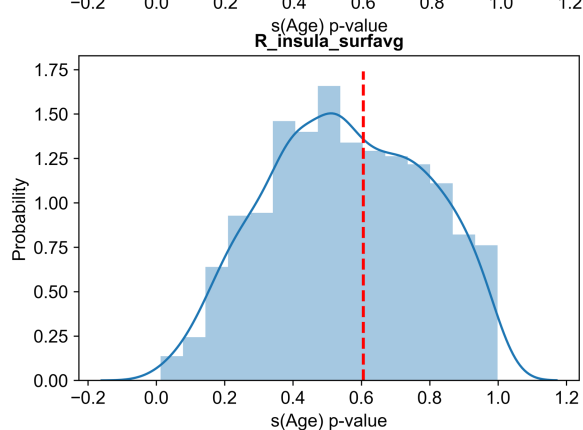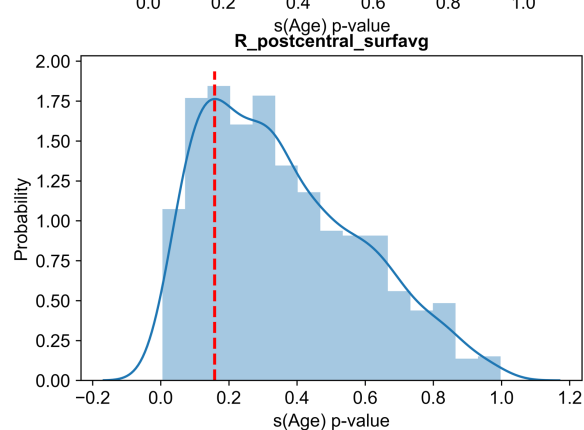

Supplement: Supplementary file 2 — Supplementary Figure S1 [file 41380_2024_2426_MOESM2_ESM.pdf]
